# Association of Polymorphisms in T-Cell Activation Costimulatory/Inhibitory Signal Genes With Allograft Kidney Rejection Risk

**DOI:** 10.3389/fimmu.2021.650979

**Published:** 2021-06-02

**Authors:** Jose Luis Santiago, Luis Sánchez-Pérez, Isabel Pérez-Flores, Maria Angeles Moreno de la Higuera, Natividad Calvo Romero, Javier Querol-García, Elena Urcelay, Ana Isabel Sánchez-Fructuoso

**Affiliations:** ^1^ Lab. Genetics and Molecular Basis of Complex Diseases, Instituto de Investigación Sanitaria del Hospital Clínico San Carlos, IdISSC, Madrid, Spain; ^2^ Immunology Department, Hospital Fundación Jiménez-Díaz, Madrid, Spain; ^3^ Nephrology Department Hospital Clínico San Carlos, Facultad de Medicina, Universidad Complutense de Madrid, IdISSC, Madrid, Spain

**Keywords:** allograft rejection, Banff classification, cytotoxic T-lymphocyte associated protein 4, kidney transplantation, polymorphism

## Abstract

The genes *CD28*, *CD86* and *CTLA-4* conform the costimulatory (CD28-CD86) or inhibitory (CTLA-4-CD86) signal in T-cell activation. T-cell immune response has a critical role in allograft rejection, and single nucleotide polymorphisms (SNPs) located in these genes have been widely analyzed with controversial results. We analyzed a group of SNPs located in the three genes: *CD28*: rs3116496; *CD86*: rs1129055; and *CTLA-4*: rs231775 and rs3087243 in a cohort of 632 consecutively recruited kidney transplanted subjects. All polymorphisms were genotyped by TaqMan chemistry and the diagnosis of rejection was confirmed by biopsy and categorized according to the Banff classification. The analyses showed a statistically significant protective effect to T cell-mediated rejection (TCMR) in carriers of the *CTLA-4* rs3087243*G allele, especially in patients with TCMR Banff ≥2 in the overall cohort and in patients without thymoglobulin induction therapy. Both associations were corroborated as independent factors in the multivariate analysis. Interestingly, associations with rejection were not found for any SNP in patients with thymoglobulin induction therapy. As expected, considering the major role of these genes in T-cell activation, no effect was observed for antibody-mediated rejection (ABMR). In conclusion, the SNP rs3087243 located in the *CTLA-4* gene may be considered a useful independent biomarker for TCMR risk especially for severe TCMR in patients who did no received thymoglobulin induction therapy.

## Introduction

The knowledge related to kidney transplantation has progressed considerably during the past years thanks to a better understanding of the role of the immune system in allograft rejection and an enhanced management of immunosuppression (1). In fact, the risk of acute rejection during the first year after transplantation is now less than 15% (2). However, rejection episodes occur and, despite all the improvements, the rates of graft survival beyond 5 years remain almost unaltered (3–5). It is well known that acute rejection after kidney transplantation is a major cause of allograft loss (6). In this complex process, that involves interaction among multiple cells, T-lymphocytes play a major role in recognizing alloantigens and, therefore, in the immune response against the donor organ (2, 7). The complete T cell activation requires the costimulatory signal through CD28 binding with CD86, which competes with the inhibitory signal through cytotoxic T-lymphocyte associated protein 4 (CTLA-4) (8). Several polymorphisms in the genes encoding these molecules have been widely studied considering the crucial significance of the costimulatory signal in T-cell activation.

The *CTLA-4* gene encodes a member of the immunoglobulin superfamily, and some polymorphisms located in this gene alter the protein expression levels (9). The SNP rs231775 (+49 A/G) in exon 1 of the *CTLA-4* gene, results in a substitution Thr17Ala and it modifies the expression of the molecule on the T-cell membrane by affecting the rates of endocytosis and surface trafficking (10–12). The protein derived from the *CTLA-4* +49*G allele has been reported to exhibit a decreased inhibitory function, based on the association of this allele with enhanced T-cell proliferation after *in vitro* stimulation (13). In addition, the G allele of the +6230 A/G SNP (rs3087243) in the 3´UTR of this gene drives low levels of messenger RNA (mRNA) for the soluble isoform of CTLA-4 (sCTLA-4). Published data suggest that sCTLA-4 blocks the CD28-CTLA-4 interaction, thereby enhancing T-cell activation (9, 14). Both polymorphisms have been studied in kidney transplantation, with controversial results ranging from association with acute rejection (15–20) to lack of association (21–23).

The polymorphism rs3116496 of the *CD28* gene in the 2q33 chromosomal region, leads to a T/C substitution at position 17 in the third intron, and it has been involved in splice site identification (20). Previous studies have reported the association between this SNP and acute susceptibility to acute allograft rejection, but the results were inconsistent, including both an increased risk (20, 24) and lack of association (18).

Finally, the polymorphisms 1057G/A (rs1129055) in the *CD86* gene causes an A304T substitution in exon 8 (25) that introduces a potential phosphorylation site in the cytoplasmic region (26).Therefore, this SNP may alter the levels of tyrosine kinase phosphorylation of the CD86 cytoplasmic tail and could influence the signal transduction pathway (20, 27, 28). Previous studies have revealed the association between the *CD86* +1057G/A variant and a reduced risk of acute rejection (29, 30), but lack of association has also been reported (31).

Given these apparently inconsistent results, we decided to analyze the aforementioned polymorphisms in a well powered cohort, in order to elucidate the potential associations with kidney transplant outcome. The identification of predictive factors involved in allograft rejection continues as an important research topic to improve long term allograft survival and to establish personalized therapies. To date, no good biomarkers exist to predict post-transplant complications (32); therefore genetic polymorphisms could be a useful tool.

## Patients and Methods

### Study Design

We performed a retrospective observational study of a kidney transplant cohort. The clinical and research activities being reported are consistent with the *Principles of the Declaration of Helsinki* considering ethical principles for human research. The study was approved by the Ethics Committee of the Hospital (CEIC Hospital Clinico San Carlos, ethical approval code 14/438-E) and written informed consent was obtained from every patient.

### Patients and Clinical Data

**Figure 1:**
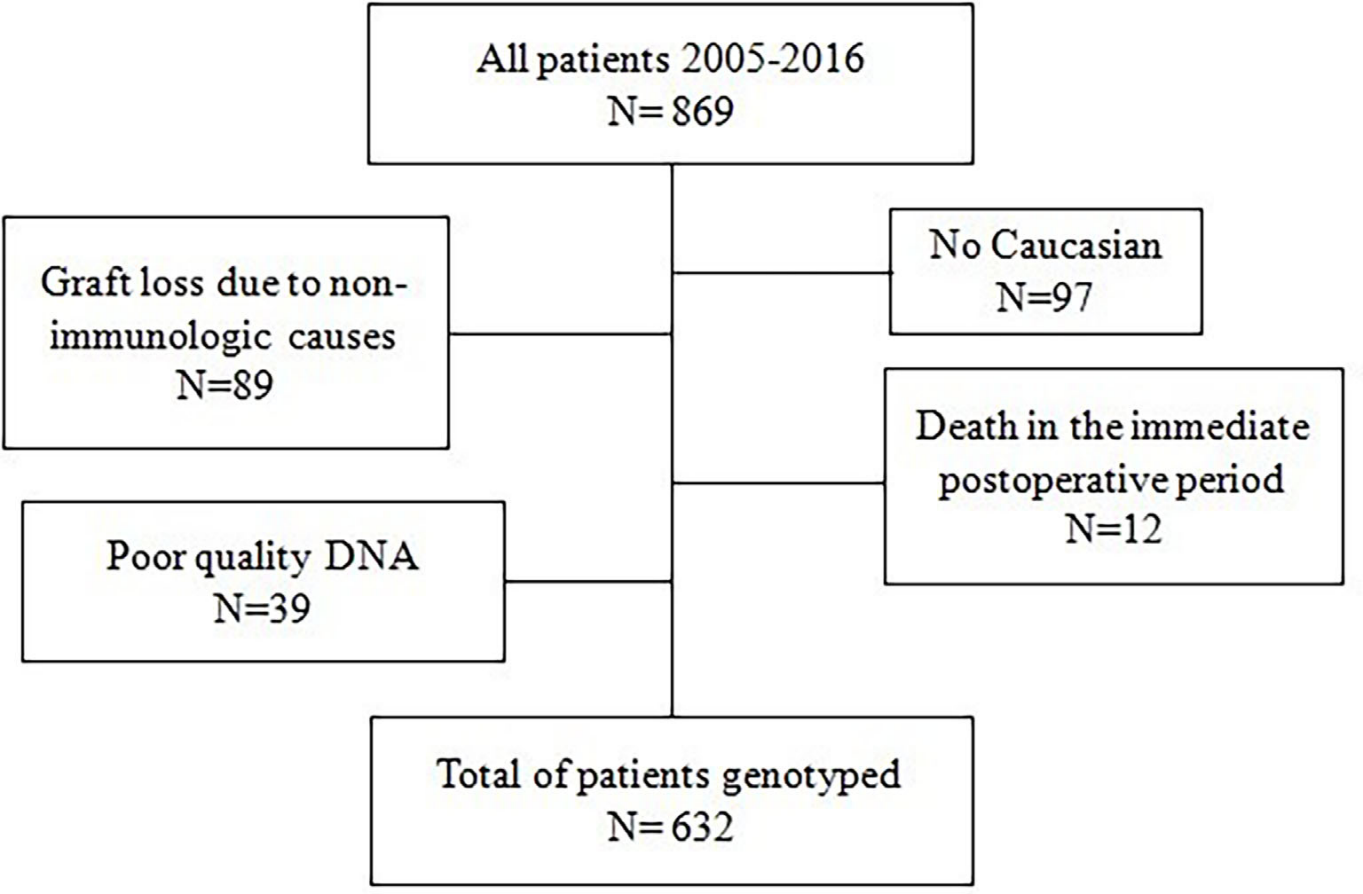
Flowchart of the patients included in the study.

Between January 2005 and December 2016 a total of 869 adult patients received a deceased donor organ in the Transplant Unit of the Hospital Clínico San Carlos (Madrid, Spain). We excluded non Caucasian patients, recipients with graft loss due to non-immunologic causes in the first three months, and patients who died in the immediate postoperative period. Our final cohort included 632 patients (**Figure 1**).

All diagnoses of rejection were confirmed by biopsy, and both T cell-mediated rejection (TCMR) and antibody-mediated rejection (ABMR) were categorized according to the Banff classification (33, 34). TCMR includes acute cellular rejection, borderline or chronic active rejections were not included. Graft loss was defined as returning to chronic dialysis or death with a functioning graft. An ultrasound-guided graft biopsy was accomplished in patients in whom acute rejection was suspected (increased serum creatinine > 0.5 mg/dl ruling out other causes of kidney function worsening) and in all patients with delayed graft function every 7 days until kidney function began to improve. Delayed graft function was defined as the need for dialysis and/or no improvement in creatinine (<1mg/dl compared to baseline) in the first week Deposition of C4d was studied by immunohistochemistry.

### Immunosuppression

The immunosuppressive protocol was as follows: patients who received a kidney from a brain dead donor were treated mainly with tacrolimus, mycophenolate mofetil, and methylprednisolone; in donors with expanded criteria or when the ischemia time was long (up to 24h), they also received Interleukin-2 receptor antagonist (IL2ra) or thymoglobulin. When the organ was donated after circulatory death, most patients received treatment with tacrolimus, mycophenolate mofetil, and methylprednisolone combined with IL2ra or thymoglobulin. In patients who received thymoglobulin, tacrolimus was introduced between days 4 and 6 after transplant. The induction therapy with IL2ra was applied in 179 patients and 350 subject received induction therapy with thymoglobulin. The group of patients treated with IL2ra was included in the non-thymoglobulin induction therapy.

### Polymorphism Genotyping

The Genomic DNA was extracted from EDTA-anticoagulated peripheral whole blood, and all polymorphisms were genotyped in a 7900HT Fast Real-Time PCR System using TaqMan assays, as recommended by the manufacturer (Applied Biosystems, Foster City, CA, USA). The assay number of each SNP was for the *CTLA-4* gene: +49 A/G (rs231775; C:_2415786_20), +6230 A/G (rs3087243, C:_3296043_10); for the *CD28* gene: +17T/C (rs3116496¸ C:25922478_10) and finally for the *CD86* gene. +1057 A/G (rs1129055, C:_7504226_10).

### Statistical Analysis

Quantitative variables were compared by using the chi-square test or Fisher exact test and expressed as frequency distributions. Qualitative variables were expressed as mean (SD) or median (IQR) for non-normally distributed variables. Variables with p<0.15 in the univariate analysis were included in the logistic regression model. The statistical package used was SPSS version 15.0.

## Results

Demographic and clinical characteristics of the kidney transplant cohort are reported in **Supplementary Table 1**. The genotypic frequencies of *CTLA-4* rs231775 polymorphisms were: 51.7% AA, 40.4% AG and 8.9% GG and for the *CTLA-4* rs3087243 polymorphism were: 24.3% AA, 51.5% AG and 24.2% GG. The genotypic frequencies of *CD28* rs3116496 SNP were 64.6% TT, 30.8% TC and 4.6% CC. Finally, the genotypic frequencies of *CD86* rs1129055 polymorphism were: 50.4% GG, 41.3% GA and 8.3% AA. The distribution of genotypes for all the SNPs studied was consistent with the Hardy-Weinberg Equilibrium (HWE) assessed by using chi-square test.

In the overall cohort (n=632), T cell-mediated rejection (TCMR) episodes were found in 21.4% of patients, the TCMR graded Banff ≥2 was observed in 16.0% and antibody-mediated rejection (ABMR) appeared in 7.1% of subjects. In this overall cohort, a protective effect of the *CTLA-4* rs3087243*G allele was found for all TCMR, and only in the dominant model for TCMR Banff ≥2 (**Table 1**).

**Table 1:** Univariate analysis for rejection.

SNP ID-Gene	Dominant model	TCMR		TCMR Banff ≥2		ABMR	
		OR (95%CI)	p value	OR (95%CI)	p value	OR (95%CI)	p value
rs231775 - *CTLA-4*	GG+AG	0.78 (0.53-1.14)	0.197	0.83 (0.54-1.28)	0.402	1.08 (0.59-1.98)	0.808
rs3087243 - *CTLA-4*	GG+AG	0.55 (0.36-0.84)	0.005	0.57 (0.36-0.92)	0.019	0.95 (0.47-1.93)	0.887
rs3116496 - *CD28*	CC+CT	0.84 (0.56-1.26)	0.410	0.81 (0.52-1.28)	0.370	0.90 (0.47-1.71)	0.742
rs1129055 - *CD86*	AA+AG	0.82 (0.56-1.20)	0.311	0.85 (0.55-1.30)	0.442	1.20 (0.66-2.21)	0.551
							
**SNP ID-Gene**	**Recessive model**						
rs231775 - *CTLA-4*	GG	0.85 (0.41-1.73)	0.644	0.65 (0.27-1.56)	0.333	1.07 (0.37-3.12)	0.784*
rs3087243 - *CTLA-4*	GG	0.80 (0.50-1.27)	0.333	0.81 (0.48-1.36)	0.425	0.67 (0.31-1.48)	0.318
rs3116496 - *CD28*	CC	0.76 (0.28-2.03)	0.579	0.60 (0.18-2.00)	0.603*	0.46 (0.06-3.41)	0.713*
rs1129055 - *CD86*	AA	1.06 (0.54-2.07)	0.872	1.57 (0.76-3.10)	0.191	1.05 (0.36-3.05)	0.788*

*Fisher exact test (two-tailed).

Next, we decided to complete the analysis stratifying our cohort by considering treatment with thymoglobulin. The stratification by induction therapy with IL2ra was not considered because this induction therapy showed increased risk for TCMR instead of protection in our cohort (63 vs 116 OR = 2.87 (1.93-4.27); p <0.001, **Supplementary Table 1**). A total of 350 out of the 632 patients (55.4%) received thymoglobulin induction therapy. In this group, 11.7% of patients suffered TCMR, 8.9% TCMR Banff ≥2, and 9.1% ABMR. In this subgroup of subjects treated with thymoglobulin, none of the polymorphisms under study showed any effect either in the dominant or in the recessive models (**Table 2**). In the other 282 (44.6%) patients who did not receive thymoglobulin induction therapy, TCMR appears in 33.3% of them; TCMR Banff ≥2 in 24.8%, and 4.6% suffered ABMR. A protective effect against TCMR was observed in carriers of the G allele of the *CTLA-4* rs3087243 polymorphism, and a trend for protection was found in patients with TCMR Banf≥2 (**Table 3**). For ABMR no association was evidenced for any of the polymorphisms in the three groups (**Tables 1**–**3**).

**Table 2:** Univariate analysis for rejection in patients with Thymoglobulin induction therapy.

SNP ID-Gene	Dominant model	TCMR		TCMR Banff ≥2		ABMR	
		OR (95%CI)	p value	OR (95%CI)	p value	OR (95%CI)	p value
rs231775 - *CTLA-4*	GG+AG	0.71 (0.37-1.37)	0.305	1.01 (0.48-2.11)	0.983	1.08 (0.52-2.23)	0.840
rs3087243 - *CTLA-4*	GG+AG	0.67 (0.32-1.41)	0.291	0.72 (0.31-1.69)	0.450	1.44 (0.54-3.89)	0.468
rs3116496 - *CD28*	CC+CT	1.21 (0.62-2.35)	0.570	1.42 (0.68-2.99)	0.351	1.00 (0.47-2.13)	0.993
rs1129055 - *CD86*	AA+AG	1.13 (0.59-2.16)	0.718	0.99 (0.47-2.08)	0.983	0.93 (0.45-1.92)	0.840
							
**SNP ID-Gene**	**Recessive model**						
rs231775 - *CTLA-4*	GG	1.13 (0.37-3.41)	0.772*	1.60 (0.52-4.92)	0.502*	1.54 (0.50-4.72)	0.508*
rs3087243 - *CTLA-4*	GG	0.96 (0.45-2.04)	0.906	1.47 (0.66-3.26)	0.339	0.66 (0.26-1.67)	0.382
rs3116496 - *CD28*	CC	0.57 (0.07-4.47)	1.000*	Undefined	0.623*	0.77 (0.10-5.98)	1.000*
rs1129055 - *CD86*	AA	0.98 (0.28-3.43)	1.000*	0.85 (0.19-3.76)	1.000*	0.38 (0.05-2.89)	0.492*

*Fisher exact test (two-tailed).

**Table 3:** Univariate analysis for rejection in patients with no Thymoglobulin induction therapy.

SNP ID-Gene	Dominant model	TCMR		TCMR Banff ≥2		ABMR	
		OR (95%CI)	p value	OR (95%CI)	p value	OR (95%CI)	p value
rs231775 - *CTLA-4*	GG+AG	0.88 (0.54-1.45)	0.613	0.81 (0.46-1.39)	0.445	0.97 (0.32-2.97)	0.961
rs3087243 - *CTLA-4*	GG+AG	0.57 (0.33-0.97)	0.038	0.59 (0.33-1.05)	0.069	0.42 (0.14-1.29)	0.198*
rs3116496 - *CD28*	CC+CT	0.73 (0.43-1.25)	0.246	0.62 (0.34-1.13)	0.119	0.59 (0.16-2.19)	0.554*
rs1129055 - *CD86*	AA+AG	0.65 (0.40-1.07)	0.092	0.75 (0.43-1.29)	0.301	2.37 (0.71-7.88)	0.148
							
**SNP ID-Gene**	**Recessive model**						
rs231775 - *CTLA-4*	GG	0.73 (0.28-1.94)	0.530	0.28 (0.06-1.24)	0.075	Undefined	0.608*
rs3087243 - *CTLA-4*	GG	0.75 (0.41-1.39)	0.363	0.57 (0.28-1.17)	0.125	0.62 (0.13-2.87)	0.739*
rs3116496 - *CD28*	CC	0.72 (0.22-2.31)	0.574	0.75 (0.20-2.73)	1.000*	Undefined	1.000*
rs1129055 - *CD86*	AA	0.94 (0.41-2.17)	0.888	1.80 (0.79-4.10)	0.160	2.93 (0.76-11.34)	0.127*

*Fisher exact test (two-tailed).

Finally, we performed a multivariate analysis for all TCMR and for TCMR Banff ≥2. We observed that the protective effect found in carriers of the *CTLA-4* rs3087243*G allele, remained as an independent factor in all TCMR and in TCMR Banff ≥2 both in the overall cohort and in the group without thymoglobulin induction therapy (**Tables 4**, **5**).

**Table 4:** Multivariate analysis for TCMR.

Parameter		*OR (95%CI)		p value
**All patients**				
Re-transplant Yes No		2.50 (1.34-4.67) 1		0.004
Male donor Yes No		1.69 (1.07-2.67) 1		0.025
PRA >50% Yes No		2.25 (1.01-5.01) 1		0.047
Thymoglobulin Induction therapy Yes No		0.18 (0.11-0.29) 1		<0.001
rs3087243 G/A- *CTLA-4* GG+AG AA		0.54 (0.35-0.85) 1		0.007
**No Thymoglobulin induction therapy**				
Recipient >60 Yes No		1.66 (0.94-2.92) 1		0.080
Male donor Yes No		1.83 (1.04-3.22) 1		0.036
rs3087243 G/A- *CTLA-4* GG+AG AA		0.56 (0.32-0.98) 1		0.042
**With Thymoglobulin induction therapy**				
Re-transplant Yes No		2.37 (1.02-5.54) 1		0.045
Male recipient Yes No		1.98 (0.94-4.19) 1		0.074
PRA >50% Yes No		2.42 (0.98-5.96) 1		0.055

*Adjusted for donor and recipient age, gender, re-transplant, cold ischemia time, Thymoglobulin induction therapy and PRA >50%.

**Table 5:** Multivariate analysis for TCMR Banff ≥ 2.

Parameter		*OR (95%CI)		p value
**All patients**				
Re-transplant Yes No		2.74 (1.39-5.40) 1		0.004
Male donor Yes No		1.66 (0.99-2.77) 1		0.054
PRA >50% Yes No		2.78 (1.15-6.73) 1		0.023
Thymoglobulin Induction therapy Yes No		0.19 (0.11-0.33) 1		<0.001
rs3087243 G/A- *CTLA-4* GG+AG AA		0.56 (0.34-0.91) 1		0.019
**No Thymoglobulin induction therapy**				
Re-transplant Yes No		3.19 (1.21-8.43) 1		0.019
Male donor Yes No		1.80 (0.95-3.40) 1		0.071
rs3087243 G/A- *CTLA-4* GG+AG AA		0.51 (0.28-0.93) 1		0.029
**With Thymoglobulin induction therapy**				
PRA >50% Yes No		5.31 (2.41-11.72) 1		<0.001

*Adjusted for donor and recipient age, gender, re-transplant, cold ischemia time, Thymoglobulin induction therapy and PRA >50%.

## Discussion

T-cell activation requires additional signals to the T cell receptor (TCR) complex, specifically costimulatory signals. Among them, antigen presenting cells display CD86, which is a ligand for two different T-cell membrane receptors, CD28 and CTLA-4 (8). Binding of CD86 to CD28 stimulates the T-cell, whereas binding of CD86 to CTLA-4 inhibits T-cell activation. Due to the important role in T cell-mediated immune response, the polymorphisms located in the genes encoding these proteins and related to their levels of expression have been widely explored in order to establish the association between these gene variants and acute allograft rejection risk. Despite the numerous studies performed to date, their results have not been consistent; in fact, the association with rejection risk is seemingly conflicting and contradictory (15–24, 29–31, 35). These apparently controversial results may be a consequence of lack of statistical power in the analyzed cohorts, since most of them included less of 200 patients.

Our data in a well-powered cohort show that among the SNPs selected, the *CTLA-4* rs3087243*G allele was associated with protection against kidney transplant rejection. The association was found in carriers of the *CTLA-4* +6230 G allele, which determines lower levels of sCTLA-4 mRNA. This finding seems to support the role of the T-cell inhibitory effect of CTLA-4 in the protection against kidney allograft rejection. The statistical power of our cohort with 632 subjects ratifies the observed protective effect, which is further confirmed by the multivariate analysis where it arose as an independent factor. Moreover, it has been reported that patients with *CTLA-4* mutations present a complex dysregulation syndrome characterized by hypogammaglobulinemia, recurrent infections derived from the immunodeficiency and multiple autoimmune diseases (36). Although these patients showed lymphoproliferation, they were lymphopenic in the periphery, with lymphocytic tissue infiltration, and the peripheral blood analysis revealed increased levels of T-regulatory (Treg) cells within CD4+ T-cells (36, 37). These evidences, together with the reported constitutive expression of CTLA-4 by Treg cells (38) as the major cell type expressing CTLA-4 (39), could explain why transplanted patients carrying alleles associated with reduced sCTLA-4 would preserve the immunosuppressive effect of Tregs, and thus would exhibit protection against acute allograft rejection.

In short, the paradigm of CTLA-4 as a major negative regulator of T cell response is under revision. It is now suggested that the mechanism of action of CTLA-4 include not only inhibitory signals, but regulatory functions, controlling the access of CD28 to their ligands (39). Thus, the alloimmune responses and the different susceptibility to rejection could be the result of CTLA-4 plasticity to perform different functions in different T-cells, mainly in Treg cells (39).

On the other hand, it is well known that the dose of immunosuppressive drugs and the immunosuppressive regimes influence the susceptibility to acute rejection (40). Randomized studies have shown that induction therapy with thymoglobulin is effective in preventing biopsy-proven acute rejection (BPAR), and specifically, steroid-resistant BPAR in kidney transplant patients, independently of other established risk factors (41, 42).

As the multivariable analysis revealed, the protective effect of the*CTLA-4* rs3087243*G allele is independent of other well-known risk factors including thymoglobulin induction. However; this therapy is so effective protecting against TCMR (only 41 patients suffered TCMR under thymoglobulin induction) that the reduced statistical power hampers the detection of the protective effect of *CTLA-4* rs3087243*G allele. The size of our cohort limits the statistical power and prevents multiple comparison corrections in the univariate analyses of the subgroups. Nonetheless, the use of a single cohort belonging to the same area of Spain is essential to genetic studies as it minimizes the population stratification. In addition, all the biopsies have been reviewed by the same pathologist, thus avoiding the bias that might occur when different observers participate to categorize rejection episodes according to the Banff classification.

## Conclusion

Our data suggest that the *CTLA-4* polymorphism rs3087243 (+6230 A/G) could be considered as a good independent predictive biomarker for kidney allograft rejection mainly for TCMR Banff graded ≥2 in patients who did not receive induction therapy with thymoglobulin.

## Data Availability Statement

The datasets presented in this study can be found in online repositories. The names of the repository/repositories and accession number(s) can be found below: https://www.ebi.ac.uk/eva/, EVA Helpdesk #381363.

## Ethics Statement

The studies involving human participants were reviewed and approved by CEIC Hospital Clinico San Carlos, ethical approval code 14/438-E. The patients/participants provided their written informed consent to participate in this study.

## Author Contributions

JS and AS-F designed the study. IP-F, MM, and NR were responsible for clinical care of the patients. JS drafted the manuscript. LS-P and JQ-G performed data collection. EU and AS-F revised the manuscript. All authors contributed to the article and approved the submitted version.

## Conflict of Interest

The authors declare that the research was conducted in the absence of any commercial or financial relationships that could be construed as a potential conflict of interest.

## Supplementary Material

Supplementary Table 1 Demographic and clinic characteristics of the kidney transplant cohort.
