# Supplementary material for: Association of Polymorphisms in T-Cell Activation Costimulatory/Inhibitory Signal Genes With Allograft Kidney Rejection Risk
Source: Front Immunol. 2021 Jun 2;12:650979. doi: 10.3389/fimmu.2021.650979 (PMC8206554; doi:10.3389/fimmu.2021.650979)
Supplement: Supplementary Table 1 — Demographic and clinic characteristics of the kidney transplant cohort. [file Table_1.docx]

**SUPPLEMENTARY MATERIAL**

**Supplementary Table 1** Demographic and clinic characteristics of the kidney transplant cohort

| Characteristic | No TCMR  (n= 497) | TCMR  (n= 135) | p |
| --- | --- | --- | --- |
| Recipient >60 years | 178 (82.8%) | 37 (17.2%) | 0.067 |
| Male recipient, N (%) | 315 (76.6%) | 96 (23.4%) | 0.095 |
| Donor >50 years | 175 (82.2%) | 38 (17.8%) | 0.124 |
| Male donor, N (%) | 346 (77.4%) | 101 (22.6%) | 0.239 |
| Donor type, N (%)  Brain death  Circulatory death  Living donors | 203 (78.7%)  289 (78.3%)  5 (100.0%) | 55 (21.3%)  80 (21.7%)  0 (0%) | 0.501 |
| Thymoglobulin induction therapy, N (%) | 309 (88.3%) | 41 (11.7%) | <0.001 |
| IL2ra induction therapy | 116 (64.8%) | 63 (35.2%) | <0.001 |
| HLA-A mismatch 2, N (%) | 239 (78.4%) | 66 (20.8%) | 0.972 |
| HLA-B mismatch 2, N (%) | 278 (77.0%) | 83 (23.0%) | 0.311 |
| HLA-DR mismatch 2, N (%) | 208 (78.5%) | 57 (21.5%) | 0.831 |
| PRA >50% | 42 (73.7%) | 15 (26.3%) | 0.339 |
| History of previous transplant (>1), N (%) | 69 (71.1%) | 28 (28.9%) | 0.051 |
| Cold ischemia time, hours [mean(SD)] | 18.01 (4.18) | 18.51 (3.61) | 0.256 |

IL2ra, Interleukin-2 receptor antagonist
